# The Role of Nonconventional Technologies in the Extraction Enhancement and Technofunctionality of Alternative Proteins from Sustainable Sources

**DOI:** 10.3390/foods14213612

**Published:** 2025-10-23

**Authors:** Cleberyanne da Silva Carvalho, Gabriela Xavier Ojoli, Mariana Grecco Paco, Nathalia Almeida Bonetti, Samantha Cristina de Pinho, Jéssica Thais do Prado Silva, Tiago Carregari Polachini

**Affiliations:** 1 Institute of Biosciences, Human and Exact Sciences (Ibilce), São Paulo State University (Unesp), São José do Rio Preto Campus, Rua Cristóvão Colombo, 2265, São José do Rio Preto 15054-000, SP, Brazil; cleberyanne.carvalho@unesp.br (C.d.S.C.); gabriela.oioli@unesp.br (G.X.O.); marianagp16@gmail.com (M.G.P.); nathalia.bonetti@unesp.br (N.A.B.); jessica.tp.silva@unesp.br (J.T.d.P.S.); 2 Department of Food Engineering, School of Animal Science and Food Engineering, University of São Paulo (USP), Pirassununga 13635-900, SP, Brazil; samantha@usp.br

**Keywords:** emerging technologies, yield, techno-functional properties, solubilization, plant-based proteins, valorization

## Abstract

In recent decades, the consumption of animal proteins has been rethought by consumers. Factors such as improved health and sustainability are key aspects of this scenario. Studies have sought innovative and sustainable technologies to improve protein extraction from alternative sources to increase their competitiveness. In this sense, the aim of this work was to combine the effects of nonconventional extraction methods on the process yield and the resulting techno-functional properties extracted from alternative proteins. The literature contains significant publications regarding the use of ultrasound (US), pulsed electric fields (PEFs), microwaves (MWs) and deep eutectic solvents (DESs) for enhancing protein extraction. Re-emerged techniques such as reverse micelles and aqueous two-phase extraction have also been reported. For this reason, the present study aimed not only to present the obtained results but also to discuss how the mechanisms associated with the aforementioned technologies impact the extraction yield and modification of proteins. In general, US tends to increase protein solubility (20–30%) and emulsifying capacity (35%); MWs can increase protein yield (25%) while reducing extraction time (50–70%); DES-based extraction tends to retain more than ~40% of the native functionality, and PEFs have demonstrated up to a 20% improvement in protein recovery. Nonconventional extraction methods have varying effects on the characteristics and quality of extracted proteins, offering benefits and challenges that should be considered when choosing the most suitable technology. The specificity related to each technology can be used to make possible interesting industrial applications involving nonanimal proteins.

## 1. Introduction

Daily protein intake and its corresponding source are some of the subjects of major concern for studies in food science and nutrition. Studies state that the general recommended daily protein intake for healthy adults aged 20–65 years is approximately 0.66 g per kilogram of body weight, which should constitute 8–10% of total dietary energy intake. This basic information underlines the importance of including protein sources in a balanced diet to ensure proper nutrition and promote health [1,2].

Another aspect to highlight is that most of the proteins consumed by adults are currently of animal origin [3]. However, in recent decades, the consumption of animal proteins has been rethought by consumers, especially those who are more concerned with animal welfare, improving health, and sustainability in the nutritional and livestock context [4,5].

Although the industries of animal-based products tend to become increasingly concerned with the welfare of the animals, there are rudimentary processing units that still make use of overcrowded environments and carry out management practices that ignore essential care. The life cycle of these animals culminates in slaughter methods that are also ethically questionable and that are somewhere allowed by law, further aggravating their suffering. The population can also be a precursor to changes in animal welfare and, consequently, excessive consumption of animal-based proteins. The consumer perspective affects decision-makers at the industrial, political, regulatory, and market levels [6].

Additionally, diets high in animal proteins may contribute to the development of chronic kidney disease by increasing the rates of decline in kidney function, insulin resistance, and albuminuria. There is also evidence that suggests an association between diets rich in animal protein and a greater risk of cardiovascular disease [7,8,9,10].

In addition, half of the greenhouse gas (GHG) emissions produced in the agricultural sector are attributed to livestock practices, which is why investments in alternative protein sectors have increased rapidly [11]. In this sense, consumers tend to decide on alternative proteins to contribute to more environmentally sustainable consumption. With this practice, they expect to reduce GHG emissions and land and water consumption, in addition to preserving biodiversity [12].

Finally, there is a growing demand for products with alternative proteins added to their formulations to improve their nutritional and technological properties [13]. This trend has boosted the market for analogs or substitutes of animal products [14,15,16]. Among the animal protein substitutes, plant-based proteins (e.g., soybean, pea, wheat), cellular proteins (e.g., microalgae), and fungal proteins (e.g., mushrooms) are worth mentioning [17]. Although there is a clear trend toward the valorization of these alternative proteins, the enhancement of process yield and how this macronutrient is affected during extraction have yet to be studied [18]. Scaling up and designing suitable processing conditions of such processes together with the efficient management of byproducts throughout the processing chain are still the main gaps to be covered, either when using conventional or nonconventional techniques. Consequently, increasing attention has been given to the development of efficient and sustainable processes for extracting these proteins.

The most commonly applied conventional extraction methods for obtaining these alternative proteins include air classification, extraction by solubilization in alkali media [19], aqueous extraction [20], ultrafiltration [21], and micellar/salt extraction [22]. Conventional processes are generally more accessible but do not always provide high yields. Additionally, they tend to use large amounts of natural resources, such as water and chemicals, in addition to requiring long processing times. However, it may lead to changes in protein structure, leading to denaturation and possible loss of functionality [23,24,25,26]. Therefore, methods that provide higher extraction yields, functionality preservation (or even intensification), lower energy consumption, and chemical use are of great interest to the Food Industry 4.0 [27,28].

Although studies have sought innovative and sustainable technologies for improving conventional processes for extracting proteins from alternative sources, their use can positively or negatively affect the technical-functional properties of proteins, thus reinforcing the need to study products and processes specifically [29]. A promising approach to meet sustainability and intensification requirements is the use of nonconventional and/or emerging technologies. Indeed, these technologies have been reported to confer more effective and sustainable protein extraction from plants [30].

**Figure 1:**
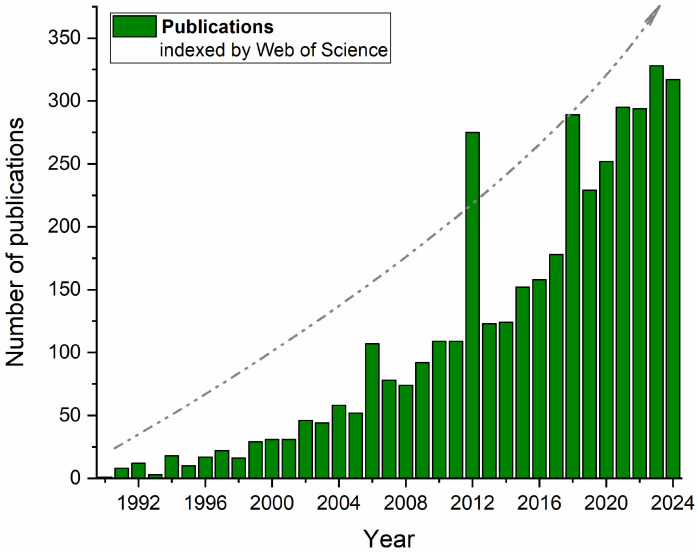
Number of publications indexed by Web of Science that include the terms “alternative proteins” and “nonconventional technologies” or “emerging technologies” over the past two decades.

Figure 1 confirms this trend when presenting the number of publications indexed by Web of Science between 1990 and mid-2024, which include the terms “alternative proteins” and “nonconventional technologies” or “emerging technologies”. In addition to the rapid increase in publications associated with predefined keywords over the years, some dispersed peaks may also be associated with particular “booms” over the years (e.g., 2006, 2012, 2017, and 2023, after the COVID-19 pandemic), when the market of plant-based and veggie foods significantly increased with new products and new technologies, especially in Western Europe.

In addition to the growing publications of nonconventional techniques for alternative protein extraction, their combination with conventional methods is often reported to generate synergistic effects on processing and product parameters [31]. Among these methods, assisted methods, as well as pretreatment, can be used to intensify conventional extraction. Jain et al. [32] quoted the use of ultrasound (US), pulsed electric field (PEF), microwaves (MWs), and deep eutectic solvents (DESs) for protein extraction as intensifying emerging approaches, among other conventional methods that have re-emerged as reverse micelles and aqueous two-phase extraction as alternatives not only to the partitioning and/or purification of proteins.

These technologies—ultrasound (US), pulsed electric fields (PEFs), microwaves (MWs), and deep eutectic solvents (DESs) are some of the most studied techniques for extracting alternative proteins from sustainable sources in the past two decades. A consolidated state-of-the-art of these technologies made it possible for the review to address established scientific evidence and, at the same time, provided strong potential for industrial application. Other methods, such as reverse micelles or aqueous two-phase systems, on the other hand, were also discussed as (re-)emerging approaches to support extraction and partitioning, as their applications are more related to purification and separation steps than to primary removal itself. Understanding the mechanisms of each technology and reviewing processing conditions contribute to the main challenges faced by the plant protein production industry: the efficient, cost-effective, and environmentally friendly use of techniques for protein extraction. Therefore, this review focused on describing specific protein extraction mechanisms, such as cell disruption, unfolding, and solubility changes, focusing on the main effects of US, PEF, MW, and DES on the extraction of proteins from nonanimal sources. Discussions of the main effects were based on processing yields and overall functionality changes.

## 2. High-Intensity Ultrasound (US)

US is a versatile technique with several uses in food processing, ranging from nondestructive analysis to homogenization and compound extraction. US can be classified as low-intensity when the intensity is lower than 1 W/cm^2^ and when the frequency is generally above 100 kHz. On the other hand, high-intensity US exceeds 1 W/cm^2^, with frequencies generally between 20 kHz and 100 kHz [33,34].

Low-intensity US, also called signal ultrasound or diagnostic ultrasound, is generally used for nondestructive testing in the areas of chemistry and biomedical analysis. High-intensity ultrasound is most commonly used for diffusion, extraction, and emulsification because of the force that the mechanical wave produces in the system, which consequently promotes rupture of structures, size reduction, and conformational change [35].

The principle of the high-intensity US-assisted extraction technique is based on the application of relatively low-frequency and high-intensity sound waves to a fluid medium, which generates physical phenomena such as cavitation, microagitation, and heating, thus promoting mass transfer enhancements.

**Figure 2:**
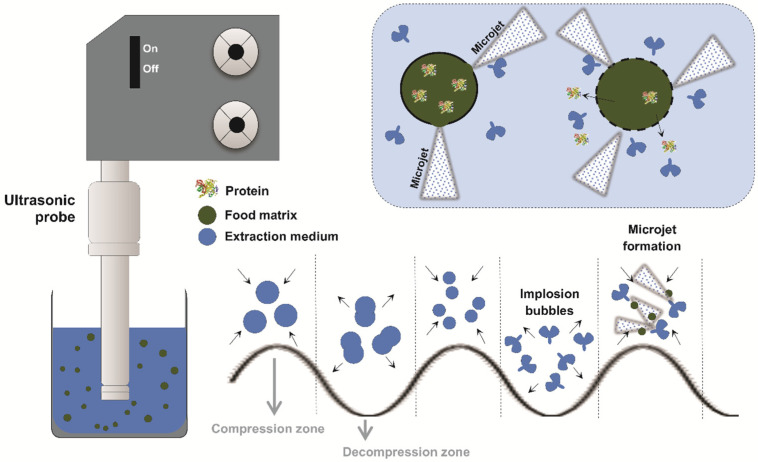
Scheme of ultrasound action during protein extraction from alternative food sources. Caption: The ultrasound probe emits ultrasonic waves that propagate through the extraction medium (blue), containing the particulate food matrix (green) and the proteins of interest (colored icon). The acoustic oscillation generates compression and decompression cycles, promoting the formation of microbubbles (cavitation). During decompression, these bubbles grow, increasing until collapse that generates high-energy microjets. These microjets directly impact the solid–liquid interface, disrupting cell structures and releasing proteins towards the extraction medium.

As exemplified in Figure 2, cavitation occurs when ultrasonic waves create alternating cycles of compression and rarefaction in the liquid, forming gas bubbles. After reaching a critical size, they implode violently and collapse among each other [36].

The implosion and collapse of the bubbles generated by cavitation release highly concentrated energy at the microscopic level, resulting in extremely high localized pressures and temperatures in the form of microjets. These effects promote the disruption of plant cell structures, intensifying processes such as protein extraction. In addition to facilitating the release of compounds, the mechanical and thermal effects of US may also cause structural changes in the extracted molecules [37].

To prevent and preserve the quality of the extracted compounds, the processing time usually does not exceed 30 min, but adjustments may be necessary depending on the matrix, solvent, equipment design, etc. [35]. However, for proteins, exposure to ultrasonic waves can be beneficial or harmful, depending on the application.

In US-assisted extraction, the nominal power usually varies between 20 and 500 W with typical frequencies ranging from 20 to 40 kHz, depending on the characteristics of the material to be extracted and the solvent employed [38,39]. Controlling the processing time is also critical to prevent sample overheating, as the energy released by the implosions of the bubbles formed by cavitation can quickly increase the temperature of the medium [40]. In many situations, the process is carried out under cooling conditions, such as in an ice bath, to dissipate the heat generated during the application of US [41].

US plays an essential role in optimizing extraction processes, providing a superior yield for recovering various compounds of interest, including proteins [40]. High-intensity US has become an important way to improve the solubility of nonanimal proteins and the extraction process itself, increasing the efficiency of the interaction between the molecules and the solvent as well as the release of proteins from the food matrix. Studies indicate that the use of US can not only increase the protein extraction yield from several alternative matrices but also improve their functionality. Table 1 compiles a series of studies that have applied USs to different nonanimal sources to obtain alternative proteins to nonanimals. A compilation of the observed effects was also included in this analysis.

When revisiting the state of the art, it is possible to determine how particular the results for each studied food source and process condition are. Even though general trends can be evidenced, studying and designing adequately nonconventional processes for a given product are needed. This gap of information is even more necessary when working with proteins, which have a heterogeneous and complex structure, composition, and functional properties, which may vary not only according to the source but also according to the handling conditions.

The need to develop suitable and more efficient processes has become even more worrying due to the trend of obtaining alternative sources of proteins—not only to meet industrial interests but also to consumers expecting healthier and affordable products. Currently, there is a growing interest in “blue proteins”, which are named proteins from aquatic sources [40,41]. However, the extraction methods, intensification possibilities, modifications, and overall effects on its functionalities need to be reviewed and discussed to identify mechanisms that may favor or increase the degree of difficulty for scientific and industrial purposes [42]. Marques et al. [31] also highlighted the use of seeds as alternative protein sources to have their extraction enhanced by sonication.

Most of the studies have focused on a fixed US frequency of ~20 kHz, with other process parameters such as the nominal power and application time varying [40,43,44]. Compared with low-intensity US, the application of lower frequencies has a potential effect on cell wall permeabilization, leading to an intensification of mass transfer and process yield [45]. Several ranges of nominal power applied were found. Most of them use nominal powers above 100 W, indicating the need for a high degree of energy to displace proteins from the plant matrix. In general, the extraction of organic compounds from plants or seeds has been based on a combination of solvent type, temperature, and/or contact technique [46], which may be enhanced by the use of US at high power levels.

**Table 1:** Recent studies on the improvement of ultrasound in the extraction of alternative proteins.

Product	Extraction Conditions	Observed Effects	References
Mulberry leaf	Frequency: 40 kHz; Power: 240 W/L; Time: 25 min.	Higher protein extraction yields compared to the conventional method. The protein yield of mulberry leaf increased by 171.76%.	[47]
Apple seed	Frequency: 16 kHz; Power not informed; Time: 30 min.	US generated impacts on structural and techno-functional properties, where ultrasonic protein exhibited increased functional properties such as emulsification, foaming, hydrophobicity, and oil absorption properties.	[48]
Defatted pumpkin seeds	Frequency: 20–25 kHz; Power: 100–300 W; Time: 10–20 min.	US had a significant effect on protein yield and recovery. The optimal conditions for extraction were 193.89 W and 19.08 min, respectively, and the predicted value of protein recovery was 79.37% and the protein yield was 48.91%.	[49]
Fava bean (*Vicia faba* L.)	Frequency: 20 kHz; Power: 57.58 W/cm^2^; Time: 20 min.	US resulted in superior results in terms of protein purity and recovery. The highest protein content is 84.09 ± 0.75% with a corresponding extraction yield of 10.94 ± 0.71%. Water, as an extraction solvent, produced significantly higher results in protein content when ultrasound was used.	[44]
Cowpea	Frequency not informed; Power: 100–200 W; Time: 5 to 20 min.	US improved the yield from 31.78% to 58.96% and from 57.26% to 68.85%. Nutritional/biological, functional, and physicochemical properties were also improved.	[50]
Camelina seed	Frequency: 40 kHz; Power: 180 W; Time: 20 min.	Significantly improved protein extraction/content and functional properties (water holding capacity, oil absorption capacity, emulsifying foaming properties, and protein solubility).	[51]
Weed (Sophia, *Descurainis sophia* L.)	Frequency: 40 kHz; Power: 180 W; Time: 20 min.	Significantly improved protein extraction/content and functional properties (water holding capacity, oil absorption capacity, emulsifying foaming properties, and protein solubility).	[51]
Watermelon seeds	Frequency: 25–40 kHz; Power: 50–200 W; Time: 15 min.	In addition to the increase in extraction (82.4%), the functional properties of the extracted proteins were also superior with US compared to conventionally extracted proteins.	[39]
Black beans	Frequency: 37 kHz; Power: 320 W; Time: 0, 10 and 20 min.	With an increase in yield of 9.70 ± 0.10, the secondary structure of the proteins is altered by US treatment, but the primary structure remains unchanged.	[52]
Lentil	Frequency: 37 kHz; Power: 320 W; Time: 0, 10 and 20 min.	With an increase in yield of 7.64 ± 0.03, the secondary structure of the proteins is altered by US treatment, but the primary structure remains unchanged.	[53]
Quinoa	Frequency: 37 kHz; Power: 320 W; Time: 0, 10 and 20 min.	With an increase in yield of 4.10 ± 0.18, the secondary structure of the proteins is altered by US treatment, but the primary structure remains unchanged.	[53]
Chickpeas	Frequency: 20 kHz; Power: 315–390 W/cm^2^; Time: 5 min.	Increase to 33.45% of protein extraction in chickpeas, where the composition of the secondary structure for chickpea protein remained unchanged.	[34]
Rice bran	Frequency: 20–35 kHz; Power: 100–200 and 300 W; Time: 10, 20 min.	Increased surface hydrophobicity, improved protein emulsifying properties, great stability, and decreased tendency with increasing US power and time.	[54]
Soybeans	Frequency: 20 kHz; Power: 315–390 W/cm^2^; Time: 5 min.	Protein extraction yields from soybean flakes increased by 68.5%. The composition of the secondary structure for soybean flakes remained unchanged.	[34]
Beans	Frequency: 20 kHz; Power: 315–390 W/cm^2^; Time: 5 min.	An increase of 16.39% was obtained when HPS of 4.5 W/cm 3 was applied, as changes in the composition of the secondary structure, causing unfolding and destabilization of the native structure of the protein.	[34]
Pecan Nut—(*Carya illinoinensis* (Wangenh.))	Frequency: 20 kHz; Power: 400 W; Time: 5 s/3 s.	The US increased the solubility of the substrate, increasing protein extraction rate (25.51%), making it easily accessible to the enzyme, thereby accelerating the chemical reaction and improving the protein yield.	[55]
Soy	Frequency: 20, 28, 35, 40, and 50 kHz; Power: 120 W; Time: 25 min.	The extraction rate improved significantly (*p* < 0.05) with the aid of US. The extraction effect was optimum at 28 kHz, and the extraction rate was improved to 73.35 g/100 g from 46.09 g/100 g control under the same condition and helps to optimize the equipment and improve efficiency.	[56]
Melon seed	Frequency not informed; Power: 300, 375, and 450 W; Time: 2.50, 5.00, 7.50, 10.00, 12.50, 15.00, 17.50, and 20.0 min.	The highest yield of 23.79% at 300 W, 31.05% at 375 W, and 28.93% at 450 W was obtained. An increase in water retention capacity, solubility, emulsion capacity, emulsion stability, and lower gelling capacity was observed in protein samples.	[57]
Turmeric powder	Frequency: 22 kHz; Power: 90 W; Time: 45, 10 min.	Maximum recovery was achieved in a minimum time of 45 min to 10 min; turmeric residue protein powder showed antidiabetic activity and maximum protein recovery (66.52%).	[58]
Walnut dregs (*Juglans regia* L.)	Frequency: 20.28 kHz; Power: 120 W; Time: 30 min.	An effective way to improve the comprehensive utilization and economic value of walnut dregs. The protein yield, purity, and CEI value of WP increased with a protein yield of 66.01%.	[49]
*Moringa oleifera* seed	Frequency: low; Power: 0, 130, 260, 390, 520, and 650 W; Time: 15 min.	Changes in secondary and tertiary structure do not significantly degrade the seed and improve thermal stability.	[59]
Pea	Frequency not informed; Power: 750 W; Time: 5, 15 min.	Higher levels of protein extraction (82.6%), shorter extraction times, and lower water consumption did not significantly alter amino acid composition, cause structural changes, and improve functional properties and biological activities.	[60]
Sesame meal	Frequency: 35 kHz; Power: 528–836 W; Time: 10, 120 min.	US increased protein yield, ranging from 41.1% to 77.7%, total phenolic content, and antioxidant capacity compared to the standard alkaline extraction method	[61]
*Cardamina violifolia* (CV)	Frequency not informed; Power: 6.5, 8.125 and 9.75 W; Time: 20, 30 and 40 min.	US treatment can increase the efficiency of protein extraction, being more effective with extract yield and purity of approximately 77%, compared to the control.	[62]
Peanut flour	Frequency: 24 kHz; Power not informed; Time: 15, 40 min.	US produced a protein extraction of 55%, obtaining 77% more protein compared to the control.	[63]
Coconut milk	Frequency: 24 kHz; Power: 6.85 W/cm^2^; Time: 2.5 min.	A higher protein yield was achieved in the US-treated samples compared to their control (49.6–86.1%).	[64]
Jackfruit leaf	Frequency: 42 kHz; Power not informed; Time: 10, 15 and 20 min.	The optimal value for protein extraction was 96.3 mg/g, exceeding the concentration by isoelectric precipitation, with the characteristic of representing a green technology.	[65]
Coffee bean	Frequency: 24 kHz; Power: 400 W; Time: 5, 15 min.	US provided higher extraction yields than those extracted by the control.	[66]
Grape seeds	Frequency: 40 kHz.; Power: 200 W; Time:15, 45 min.	US-treated albumin fractions showed higher solubilities, emulsifying properties, and in vitro digestibilities, but lower water binding capacities and thermal stability.	[67]
Bunch beans (GPI)	Frequency: acima de 20 kHz.; Power: 150, 250, and 350 W; Time: 5, 15 min.	US resulted in significant structural modifications and enhanced functional properties of guar protein isolate (GPI). A decrease in the particle size compared to native GPI (control) was also observed.	[68]
Silkworm (*Bombyx mori*)	Frequency: 40 kHz.; Power: 500 W; Time:15, 30 min.	US induced changes in the protein conformation that improved the emulsification and foaming properties. US also increased the antioxidant activity of this protein.	[69]
Sugar maple leaves (SML)	Frequency: 20 kHz.; Power: 500 W; Time:15 min.	US homogenization pretreatment improved protein extraction yield, temperature of protein denaturation was increased together with the functional and antioxidant properties.	[43]
Grape seeds	Frequency: 40 kHz.; Power: 200 W; Time: 60 min.	US showed an improvement in the protein extraction yield and overall stability.	[70]
Aquatic plant Duckweed (*Lemna minor*)	Frequency: 20 a 100 kHz.; Power: 400 W; Time: 20, 30 min.	US generated changes in the color, structure, and FTIR spectra of the obtained concentrates, which resulted in improvements in the solubility, emulsifying properties and foaming capacity.	[40]

In a study of protein extraction from soybean seeds, for example, US-assisted extraction at an input power of 1280 W in 500 mL (2.56 W/mL) increased the extraction yield by approximately 46% compared with that of nonsonicated treatments [71]. The resulting sonicated soy protein isolate had an increase in solubility of 34% at pH 7.0, probably due to the reduction in the particle size of the isolate. In general, sonication slightly decreased the emulsifying and foaming capacities by up to 12% and 26%, respectively. This could be related to alterations in protein conformation that increase the difficulty of unfolding at the interface.

In Spirulina extracts, the protein extraction yield was increased by up to 136% with US compared with that of the treatments without US, mainly due to the effective disruption of microbial cell walls. This finding reinforces that US is a powerful tool for optimizing protein extraction, especially in matrices with thicker or hard-to-break cell walls [72]. Moreover, the in vitro digestibility of the obtained proteins increased 2-fold when US was applied. In terms of some techno-functionalities, the water holding capacity (WHC) and emulsifying and foaming capacities were also improved when the materials were extracted with the assistance of US.

The protein content of mulberry leaf protein concentrate extracted by US-assisted cellulase degradation was the highest, at 62.69%, in comparison with the 51.41% obtained by conventional alkaline extraction and acid precipitation and the ~59% obtained by conventional cellulase degradation. Microstructural analysis of mulberry leaf proteins demonstrated that US-assisted cellulase extraction can break down the dense structure of protein in the raw material, reducing the average size of proteins and increasing the specific surface area and roughness. These results may be a consequence of the decrease in the α-helix content of the proteins extracted when US was used. On the other hand, the β-turn and random coil contents of the proteins increased under these conditions as a consequence of the unfolding of the α-helix structure to form new β-sheet and random coil structures. The particular functional characteristics of the proteins extracted with the assistance of US were highlighted. Compared with the conventional method, it presented the highest values associated with the highest solubility, which is interesting for food applications [47].

When evaluating the changes in the structural properties of legume proteins extracted after US exposure, Quintero-Quiroz et al. [53] reported that alterations in the secondary structure of proteins, such as α-helices and β-sheets, have a direct impact on further Maillard reactions. US causes partial unfolding of the protein structure, exposing functional groups such as amino acid free radicals. For example, the exposure of ionized acidic amino acids, such as glutamic acid and aspartic acid, as well as leucine and glycine, intensifies interactions with reducing sugars. Given the large number of food applications involving the Maillard reaction, sustainable techniques to unfold proteins may serve to valorize protein ingredients and produce clean-label products. This valorization is due not only to the Maillard reaction but also to the increase in the reactivity of the amine groups, which consequently improves the solubility, foaming and emulsifying capacity, hydrophobicity, and oil-holding capacity of protein isolates [49].

In the work of Byanju et al. [34], the authors reported a significant increase in the extraction of proteins from chickpeas with the use of US. The application of US reduced the proportion of α-helix structures, whereas the β-sheet structure increased or remained unaltered in some cases. In addition, there was a significant increase in the fraction of disordered structures, indicating a loss in the native conformation. This phenomenon results in a more flexible and open configuration, exposing free sulfhydryl (–SH) groups and forming different protein conformations in the same concentration/isolate.

The resulting effects of US on the structural and techno-functional properties of proteins also impact further food applications. In general, proteins subjected to the sonication process tend to exhibit increased emulsification capacity, foaming capacity, hydrophobicity, and oil holding capacity. These observations were, in fact, evidenced by Gani et al. [48] when studying proteins from apple seeds. A similar trend was also reported when proteins extracted from camelina seeds and from bitter melon seeds were evaluated [51,57].

Therefore, the US-assisted extraction process can be used as an effective technique to intensify the extraction of proteins from alternative sources, especially those with more rigid and difficult-to-rupture cells [48]. Sonication is also interesting for the food ingredient industry because it is able to unfold the dense structure of proteins, thus reducing the average size of the protein chains and increasing their specific surface area and roughness. These results might improve the solubility and different techno-functional properties of interest for food applications.

## 3. Pulsed Electric Field (PEF)

Pulsed electric fields (PEFs) constitute another emerging technology in food processing that consists of applying high-intensity short-duration electrical pulses between two electrodes. The electrical current promotes electroporation, which temporarily or permanently increases the permeability of cell membranes. The creation of these pores facilitates the transport of molecules and ions through cells or tissues toward the solvent [73].

**Figure 3:**
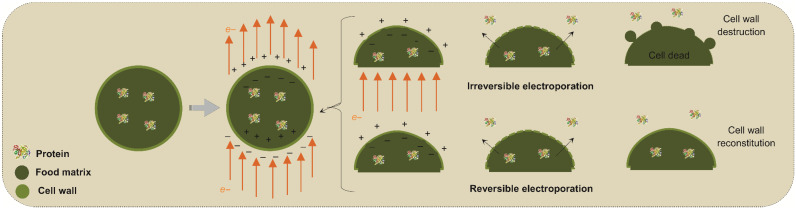
Temporally and permanent electroporation during pulsed electric field application in plant cell membranes.

Figure 3 shows a schematic of the mechanism of actuation of PEFs on cell membranes and even in the cell wall in the case of plant-based sources. In this scheme, the PEF application through the conduction medium and cell membranes orients the charges along with the electrical field, thus creating opposite charges on the inner and outer sides of the phospholipidic bilayer membranes and wall cells. As the electrical strength becomes more intense and to promote neutralization, the opposite charges tend to compress the membrane until some ruptures occur along the surface. As a result, by opening pathways to increase the mass transfer rates of intracellular compounds and the entry of solvents into the cells, the release of cellular components such as proteins into the extracellular medium is improved, resulting in interesting extraction yields [74,75].

After these pores are created, the original structure of the membrane can recover if the applied electrical field is not intense; thus, this phenomenon is referred to as temporal electroporation. This effect is interesting for treatments in which the structure, quality and/or vigor of the material source needs to be preserved. In a nondestructive way, this technology preserves sensory and nutritional characteristics while increasing mass transfer. In some cases, even after the electric field has been disrupted, the pores (permeabilization) may remain for a few minutes, allowing the cell to return to its original state afterwards [76].

In contrast, if the electrical field is intense enough, these pores can be permanent to promote a permanent electroporation phenomenon. This latter is interesting for microbial inactivation if no commitment of the food structure is observed. On the other hand, one of the limitations of PEF for protein extraction is that, when using high electrical intensity for obtaining high cell disruption rates, the quality of the extracted fractions may be affected by also altering the technological and functional properties of the proteins while spending high input energy [77,78].

However, the extraction of compounds from the food matrix, in which the occurrence of damage in the matrix is indifferent, is also desirable. For example, Gateau et al. [79] studied the use of PEF in the extraction of proteins from microalgae as a nonconventional technology to obtain high-quality proteins from sustainable sources. Because the residue of the protein extraction may serve as a source of carbohydrates for conversion into bioproducts, it is also interesting to have a disrupted and pretreated biomass for further biorefinery applications. In fact, when alternative sources of proteins are used, the structure of the residue extracted is not highly important but rather interesting because of its circular bioeconomy.

This technology was originally studied for use against microorganisms [80], and its use continues even in the most up-to-date publications [81]. In recent years, an increasing number of studies have focused on the application of this technique for the extraction of intracellular compounds of interest, such as bioactive compounds, lipids, and proteins [74]. For this, the intensity of the electric field must be greater than a critical value to cause electroporation. Consequently, the resulting effect varies according to the cellular matrix and the main focus of the process [82,83].

In general, as reported by Chudasama et al. [84], the extraction yield of compounds of interest tends to be enhanced when working with higher electric field strengths. Shorter pulses are more effective in temporary permeabilization by reversible electroporation. As a consequence, the application of more pulses per time period (high frequency) increases the intensity of the treatment. On the other hand, enhancing the overall intensity of the treatment may result in undesirable thermal effects for heat-sensitive products. For protein extraction, an intensity between 1 and 5 kV/cm is usually reported for both plant and animal cells, although higher values can be used for more rigid matrices [79,85]. The configuration of the duration of the pulses is crucial for extraction and is used between 1 and 100 μs for sensitive proteins. In addition to pulses of shorter duration, the use of 10 to 100 pulses tends to have a better effect on the preservation of the protein structure. The repetition rate of the pulses can also vary from 1 to 10 Hz at controlled temperatures of 20 to 30 °C to avoid thermal degradation of the proteins and enhance extraction.

These parameters can be managed depending on the matrix source, the desired yield of extraction, and secondary effects on the extracted proteins [62,75,86]. In the case of plant-based sources, the application of high-intensity electric fields in the form of short-duration pulses (μs) is recommended to disrupt cell walls and increase protein extraction [87,88]. In this sense, Table 2 compiles a series of studies that used PEF for enhancing protein extraction from different sources, alternatively to animals.

When focusing on the use of PEF to extract protein from alternative sources, Kamboj et al. [86] reported an increase in the protein content and antioxidant activity of perilla seed meal (PSM) residue from the perilla oil extraction industry, a plant native to Southeast Asia. Compared with conventional alkaline extraction, PEF-assisted alkaline extraction had a positive effect on the protein content (up to 10.58%) of the concentrate obtained from PSM. On the other hand, the authors also reported that increasing the number of pulses beyond 10 is unnecessary and may also cause negative results. Increasing the pulses and electrical strength from 6 kV/cm to 9 kV/cm also decreased the yield. The authors associated this behavior with possible protein degradation and/or chemical modifications that increased the difficulty of separating soluble proteins/peptides during extraction.

**Table 2:** Recent studies on the improvement of the pulsed electric field in the extraction of alternative proteins.

Product	Extraction Conditions	Observed Effects	References
Yeast *S. cerevisiae*	Frequency: 39.8 and 159.3 Hz; Intensity: 10, 15, and 20 kV/cm; Time: 50–200 μs.	Extraction of intracellular compounds such as mannoproteins and β-glucan.	[75]
Perilla Seed Meal (PSM)	Frequency not informed; Intensity: 3–15 kV/cm; Time: 10–150 μs.	From 6 kV/cm there was a decrease in the content of extracted proteins.	[86]
White mushrooms	Frequency: 400 Hz and 800 Hz; Intensity: 2.4, 24.8, and 38.4 kV/cm; Time: 69.4 and 136μs.	The combination of PEF with light heating resulted in a significant increase in protein extraction.	[88]
*Cardamine violifoli*	Frequency: 1.01 kHz; Intensity: 6.67 kV/cm; Time: 20 min.	Obtaining selenoproteins with 57% purity.	[62]
Microalgae (*Chlorella vulgaris*)	Frequency: 20 kV/cm; Intensity: not informed; Time: 50 μs.	Extraction of free protein up to 29% without impeding cell growth.	[89]
*Porphyridium cruentum*	Frequency: 0.5 Hz; Intensity: 1.56–7.26 kV/cm; Time: 2.2–7.2 μs.	It selectively avoided the release of the protein calmodulin (a food allergen).	[90]
Olive pomace	Frequency not informed; Intensity: 1.0–6.5 kV/cm; Time: 15 μs.	PEF as a pretreatment doubled the protein concentration and decreased the extraction time.	[91]
Sesame pie	Frequency: 0.5 Hz; Intensity: 13.3 kV/cm; Time: 10 μs.	Significant improvement in protein extraction when used as pretreatments.	[92]
Tomato waste	Frequency: 20 Hz; Intensity: 0.5–2.5 kV/cm; Time: 15 μs.	The concentration of protein was released as the strength and time of the electric field increased.	[82]
Mixtures of grasses (70%) and clover (30%)	Frequency: 305 Hz; Intensity: 1.1 kV/cm; Time: 5 μs.	When used together with alkaline pretreatment, it released insoluble chloroplast membrane proteins.	[93]
Oats	Frequency: 1.01 kHz; Intensity: 6.67 kV/cm; Time: 60, 90–120 min.	The results suggested that the oat protein extracted by the ChCl-1,4-butanediol/water binary mixture had higher protein content, solubility, foaming capacity, and stability.	[94]
Pea	Frequency: 400 Hz; Intensity: 1.65 kV/cm; Time: 5 min.	PEF was able to modify the structure of the protein by inducing unfolding, intramolecular rearrangement, and aggregate formation. These results suggest the potential of PEF to guide the structure of proteins and improve their technological functionality.	[95]
Rice	Frequency: 400 Hz; Intensity: 1.65 kV/cm; Time: 5 min.	PEF was able to modify the structure of the protein by inducing unfolding, intramolecular rearrangement, and aggregate formation. Structural changes were associated with negligible changes in functional properties.	[95]
Gluten	Frequency: 400 Hz; Intensity: 1.65 kV/cm; Time: 5 min.	PEF was able to modify the structure of the protein by inducing unfolding, intramolecular rearrangement, and aggregate formation. However, these effects were strongly dependent on the nature of the protein and pH.	[95]
Seaweed (*C. vulgaris*)	Frequency: 4.5 Hz; Intensity: 40 kV·cm ^−1^; Time: 1 μs.	It induces irreversible permeabilization of the membrane and, consequently, programmed cell death. The release of proteins is likely facilitated by autolytic processes associated with programmed cell death.	[96]
Papaya peel	Frequency not informed; Intensity: 40–10 kv; Time: 2720 s ≈ 3/4 h;	The proposed two-stage PEF method allows a significant increase in the yield of high-value-added compounds and in the antioxidant capacity of papaya peels, even at neutral pH, and does not require the use of additional chemical compounds.	[97]
Residual brewer’s yeast	Frequency not informed; Intensity: 10 kV/cm; Time: 10 min.	The PEF method was a promising new method for extracting protein from brewer’s yeast residues that will benefit the food and agricultural industry	[98]
Green algae Lettuce (*Ulva lactuca*)	Frequency not informed; Intensity: 7.5 kV/cm; Time: 0.05 μs.	Protein yield reaches 15.1%. This study reported the highest protein (~39%) and carbohydrate (~51%) yields of the four technologies utilizing high-shear homogenization.	[99]
Soy	Frequency: 2, 8 and 500 Hz; Intensity: 0–40 kV/cm; Time: 0–547 μs.	Solubility and hydrophobicity increased with increasing PEF strength and treatment time at constant pulse width.	[100]
*Nannochloropsis* Microalgae	Frequency not informed; Intensity: 20 kV/cm; Time: 10.0 ± 0.1 μs.	PEF allowed for the selective extraction of a few different pure proteins. The pretreatment of PEF with the parameters used in this study was ineffective for pigment extraction.	[101]
Rapeseed stems and leaves	Frequency: 2.0.5 kHz; Intensity: 20 kV/cm; Time: 1 μs.	The results of this study show the efficacy of PEF treatment as a new way of valuing rapeseed stems and leaves. Polyphenol and protein extraction yields can be highly increased.	[102]
Large green algae *Ulva ohnoi*	Frequency: 3 h; Intensity: 1 kV/cm; Time: 50 μs.	In addition, PEF combined with pressing increased the protein of coextracted macroalgae by more than 4 times and the ash by 1.5 times compared to pressing alone.	[103]
Green marine macroalgae *Ulva* sp.	Frequency: 247 kJ/kg; Intensity: 50 kV/cm; Time: 50 μs.	It resulted in a ∼7-fold increase in the total yield of protein extraction compared to conventional.	[104]
Chickpea seeds	Frequency: 100 Hz; Intensity: 0.7–0.8–0.9–1.0 and 1.1 kV/cm; Time: 30–60–90–120 and 150 s.	PEF efficiently contributed to obtaining chickpea protein isolates with excellent functional properties, making possible the production of proteins with highlighted functional properties.	[105]
Wet biomass of A. platensis (*Spirulina*)	Frequency: 20 Hz; Intensity: 7.8 kV/cm; Time: 0.113–0.565–2.260 and 3.955 μs.	PEF demonstrated significantly accelerated protein extraction from Spirulina, while positively affecting the purity of the extract. A synergistic effect of electroporation and proteolytic enzyme activity during incubation of PEF-treated biomass was also observed.	[78]
Defatted yellowworm flour (*T enebrio molitor*).	Frequency: 20 Hz; Intensity: 1.5–3.125 and 5 kV/cm; Time: not informed.	A 27% increase in protein yield and decreased molecular weight were observed when comparing to the control extracts. Furthermore, PEF induced modification of the protein structure, as indicated by a reduction in fluorescence and β-sheet content. The observed changes had an impact on the measured foaming properties,	[106]
Green marine macroalgae (*Ulva* sp.)	Frequency: 5–10 Hz; Intensity: 0–750–1000 and 1250 Vcm^−1^; Time: 60 μs.	Greater efficiency and especially environmental sustainability, as no large waste streams are generated. It demonstrates superior extraction performance to the conventional alkaline extraction. In addition, the values for water and oil holding capacity showed suitability for various food and biochemical applications.	[107]

Buchmann et al. [89] studied the use of PEF in *Chlorella vulgaris* cultures. The authors reported that as the intensity of the electric field increased, the rate of protein extraction also increased. However, the increase in extraction rates caused undesirable growth of microalgae due to the presence of free protein extraction, indicating that the efficiency of PEF extraction without affecting the vitality of the microalgae is probably due to temporal electroporation. Finally, the authors reported that the extraction efficiency depended on the cell growth phase, which was limited mainly by diffusion due to the biomass loading in the extraction medium.

Another study reported a permeabilization of 53.8% of the *Porphyridium cruentum* population (6 kV/cm by 90 μs), which resulted in the solubilization of approximately 97% of the water-soluble proteins (β-phycoerythrin) after 48 h of extraction [108]. Although most studies have shown that PEF increases the yield of protein extracted from microalgae, comparative studies suggest that other unconventional methods should be studied [109].

A study conducted by Andreou et al. [82] revealed that using PEF as a pretreatment in olive pomace (1.0 to 6.5 kV/cm, 0.9 to 51.1 kJ/kg, pulse of 15 μs) followed by conventional solid–liquid extraction (50% ethanol–water solution) doubled the concentration of protein in the dispersant, in addition to decreasing the extraction time. In another work carried out by Andreou et al. [91], the efficiency of PEF pretreatment (5 kV/1.5 ms) in tomato waste was evidenced by the increase in protein extraction from 88.9 mg/100 g of tomato waste (untreated) to 145.1 mg/100 g of tomato waste.

Sarkis et al. [92] also applied this technology as a pretreatment for sesame cake, which resulted in the best extraction under an energy input of 83 kJ/kg. The authors suggest that when a PEF is used followed by hot extraction, it is possible to reduce the diffusion temperature. This can be beneficial, as 20 min of extraction at 60 °C can decrease the protein concentration due to denaturation and coagulation.

Recent studies have diversified the use of the PEF technique. Steinbruch et al. [107] reported the intensification of aqueous extraction of protein from fresh biomass of the green alga *Ulva* sp. The authors also highlighted the performance of extraction together with its environmental sustainability without generating large amounts of waste. The extracted proteins presented increased water- and oil-holding capacities, suggesting interesting food and biochemical applications.

PEF application (6.67 kV/cm, 1.01 kHz) was used to increase the yield of protein extracted from *C. violifoli*. In addition, selenoproteins with 57% purity were obtained. They are able to incorporate selenium directly into their structure during protein synthesis, in addition to presenting antioxidant functions. Even though there was an increase in protein extraction compared with conventional extraction, PEF conditions were not optimized and, consequently, were not as efficient as ultrasound application in a comparative study. However, the quality and structure of the proteins in the PEF-based extracts were similar [62]. The application of PEF followed by ultrasonication did not result in greater results than did ultrasound-assisted extraction, probably because PEF was able to release proteins into the extraction medium and leave them exposed to ultrasound, increasing the likelihood of denaturation and decreased solubilization.

In view of that, PEF presents advantages by involving less intense heating effects than those of ultrasound application and even some conventional methods. This minimizes the risk of denaturation and maintains the secondary and tertiary structure of the proteins, which may be interesting for food applications and nutrition. Some applications can require functional proteins with excellent structural properties [105].

Indeed, PEF application also impacts the structural and techno-functional properties of proteins. Under well-designed conditions, PEF tends to improve the solubility of the extracted proteins, a crucial property for improving their interaction with other components and their extractability. The exposure of functional sites together with the preservation of the native structure can also improve emulsification capacity and gel formation. Proteins extracted by PEF often have good foaming properties, which benefits products such as milk and beverages. In general, PEF is recognized by preserving the functionality and integrity of proteins [110].

Notably, when alternative sources of proteins are used, the allergenicity of the extracted fraction should be considered. Prabhu et al. [103] applied PEF pretreatment combined with a pressing technique on *Ulva ohnoi* seaweed to obtain a protein concentrate, among other components, which has the potential to present protein sequences annotated to allergens. The concentration of proteins extracted was four times greater than that extracted by pressing alone. Polikovsky et al. [90] studied the individual application of PEF to *Ulva ohnoi* but also focused on the allergenicity of the resulting fraction. In addition, compared with osmotic shock treatment and mechanical pressing, the application of 50 PEF pulses (7.26 kV/cm, duration 2.3 μs, 0.5 Hz) was the most efficient technique for extracting proteins, and compared with the control, the application of PEFs selectively prevented the release of calmodulin (annotated to allergens) without PEFs.

The use of PEF as a pretreatment has been shown to be a potential technology to increase the yield and reduce the time of protein extraction from nonanimal sources. With respect to the impact of PEF technology on the extraction of protein from microalgae, this technology has proven to be a potential substitute for conventional methods and is faster than some nonconventional treatments. The effect of PEF on the techno-functional properties and quality of the extracted proteins is challenging and is of interest for food applications [111].

In brief, PEF has emerged as an innovative and efficient technology for enhancing protein extraction, especially from plant sources and microalgae in place of meat sources. This method has advantages such as increased extraction yield, preservation of sensory and nutritional properties, and improved techno-functional properties. In recent years, PEF has been recognized as an efficient technology for the cascade extraction of a series of compounds, which makes it possible to obtain a variety of valuable biomolecules from biomass, such as spent *S. cerevisiae* yeast [112]. Although the impact of processing conditions on the efficiency and quality of extraction still requires further study, agro-industrial coproducts could be valorized by encouraging PEF application in cascade applications, which would also contribute to the circular economy strategy [105,110].

## 4. Microwave (MW)

**Figure 4:**
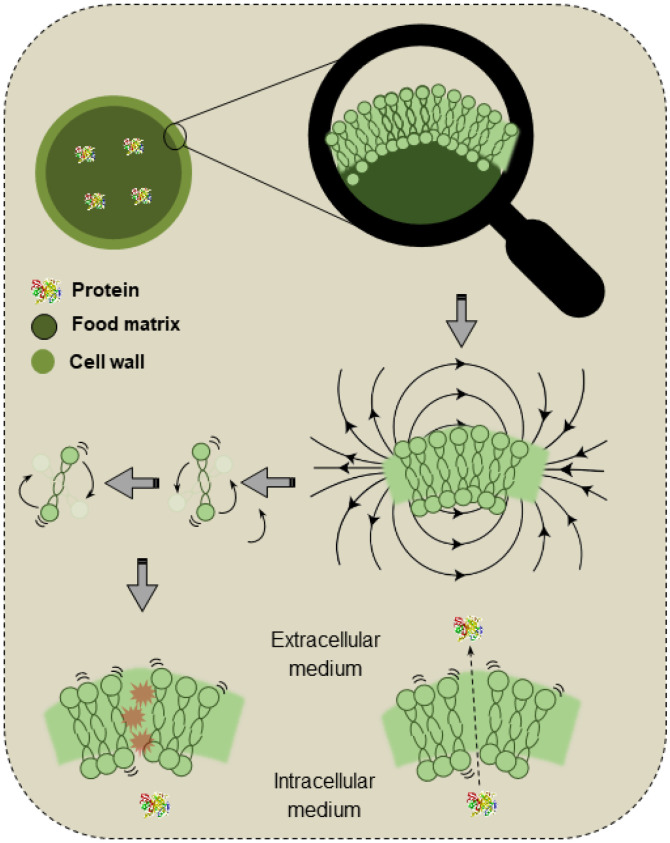
Schematic demonstration of the effects of microwaves on the cell walls and membranes.

Microwaves are electromagnetic waves with frequencies varying from 300 MHz to 300 GHz, a range between radio frequency and infrared waves. These waves are generated by the action of a magnetron, a device responsible for converting electrical energy into microwaves. The generated waves are conducted to the cavity of the instrument, where the sample to be treated is placed. The sample absorbs energy due to ionic polarization and dipole interactions, which results in heating [113]. Figure 4 exemplifies the mechanisms of MW actuation on the cell walls and membranes.

MWs selectively interact with polar molecules and induce intracellular heating, with the intensity varying according to the dielectric properties of the material. The “hot points” and localized pressure in the cell walls lead to cell rupture, improving the extraction of intracellular compounds such as proteins. As the applied power increases, a significant reduction in the extraction rate is also expected [114].

In comparison with the conventional method of protein extraction by alkaline solubilization, the extraction assisted by MW presents several advantages: the extraction process is considered a green technology, because it does not involve the use of pure solvents, causing a reduction in solvent use of 10–33% [115,116], besides demanding lower amounts of water and energy [117,118,119]. Another differential is the use of short treatments, varying between 30 s and 10 min, also resulting in high rates of extraction. When compared with other conventional and nonconventional methods, MW will achieve a 50% reduction in CO_2_ footprint during life cycle assessment (LCA), reinforcing the affirmation of the production of more sustainable proteins [120,121].

When extending microwave radiation, rapid heating of the sample is notorious, leading to protein unfolding or partial denaturation [122]. The study of Z. Li et al. [123] reports protein denaturation levels of more than 20 to 40%, compared to ultrasound at moderate intensity, which yields denaturation of 15 to 30%. However, PEF, under equivalent conditions, yields protein denaturation of 30 to 60%. Unlike ultrasound and PEF, which induce mechanical effects, the thermal effects of microwave radiation are pronounced even at low temperatures and treatment duration [122,123].

At both the industrial and laboratory scales, the frequencies of MW applications commonly range from 2.45 GHz to 3.00 GHz. Concerning the input power applied, the literature reports a wide range of values comprising powers from 100 to 750 W. The nominal power applied depends on the robustness of the matrices, the sensitivity of the extracted proteins, and the degree of modification/denaturation accepted. For more complex structures (which are alternative sources, such as plant-based coproducts and seeds), higher values are used to promote access to the intracellular contents and inner matrices. However, it can cause changes in the structure of proteins [124,125], highlighting the specific need to adequately study the process to balance the efficiency and quality of the resulting product.

MW extraction can generate undesirable changes in the structural conformation if it is not performed under well-designed operating conditions. With rapid heating and/or the use of too much power, modifications may occur in the secondary, tertiary, and even quaternary structures of the proteins. The loss of secondary structures, such as alpha-helices and beta sheets, can expose previously protected functional groups, such as amine or carboxyl groups. On the other hand, this structural alteration can increase the reactivity of proteins and their solubility [126].

Studies with promising results on alternative and sustainable sources of proteins have been reported with the assistance of MW technology. For the MW-assisted extraction of proteins from peanuts, a power of 725 W was applied for 8 min. As a result, 55% protein extraction resulted in recovery, which was 77% greater than that of the extraction control without MW application. For the extraction of soy protein, a frequency of 2450 MHz was applied for 30 min at 60 °C [63]. In these experiments, an increase of 58% in the extraction yield was obtained compared with that of the conventional process with hot water [127,128].

Barrios et al. [114] performed MW extractions for more efficient protein recovery from agri-food waste, such as brewer’s spent brewer grains (BSGs), spent coffee grounds (SCGs), and kale stems. The extraction yield in alkaline media varied for each source used, reaching a minimum value of 61% and a maximum value of 96%. In addition, low concentrations of NaOH (0.5 to 1.3 M) and time intervals (3 to 15 min) were required for the processes analyzed. For the specific case of kale stems, the authors highlight that hot water may provide interesting results for extracting proteins with the assistance of MW. This finding reinforces that, for a given material, MW can not only improve the extraction rate but also reduce chemical usage.

Martínez-Padilla et al. [64] reported the results of nonconventional technologies used to extract coconut oil and proteins from coconut pulp. Coconut milk was prepared, and MW (1 min, 3 pulses of 20 s; 2.5 GHz; 4.31 kW/kg by pulse) was applied to the milk prior to destabilization at pH 4 to obtain a cream and a protein-rich aqueous fraction. In addition to improved oil recovery compared with the control, up to 58% more protein could be recovered in the protein-rich fraction obtained from coconut milk.

When the protein extraction process from rice bran was studied by Bedin et al. [129], the authors obtained yields similar to those of the conventional extraction method but 30 times faster when MW technology was used as an assistive technology. The most favorable conditions for the extraction of 79.98% of the protein were 55 °C and pH 11 for 120 s [130].

Table 3 presents a compilation of studies together with the conditions used to report the use of MWs for the extraction of protein from alternative sources. In brief, the MW has been shown to be a promising alternative for the extraction of vegetable proteins because of its efficiency, intensification properties, and lower environmental impact. In addition, the MW tended to preserve the functional properties of proteins when used under controlled conditions. On the other hand, with the use of high power and long exposure times, structural changes may occur.

**Table 3:** Recent studies on the improvement of the use of microwaves in the extraction of alternative proteins.

Product	Extraction Conditions	Observed Effects	References
Peanut	Frequency: not informed; Power: 725 W; Time: 8 min; Temperature not informed.	It extracted 100% protein with 55% yield compared to the conventional extraction method.	[63]
Soy	Frequency: 2450 Hz; Power: not informed; Time: 30 min; Temperature: 60.1 °C.	58% increase in extraction compared to hot water.	[128]
Rice bran	Frequency: 50–60 Hz; Power: not informed; Time: 120 s; Temperature: 55 °C.	79.98% protein content of rice bran. 30 times faster than the conventional extraction method.	[129]
Rice bran	Frequency: 2450 MHz; Power: 600–1000 W; Time: 60–120 s; Temperature not informed.	The protein yield was higher than that obtained from an alkaline extraction. Protein digestibility was increased.	[131]
Rice bran	Frequency: 2450 MHz; Power: 800 W; Time: 20, 30, 40, 60 and 90 s; Temperature not informed.	82.6% protein recovery.	[132]
Beer fermentation bagasse	Frequency 50 Hz; Power: not informed; Time: 9.98 min; Temperature: 110 °C.	93.99% of the protein extraction yield from used beer grains.	[114]
Coffee Powder	Frequency 50 Hz; Power: not informed; Time: 3.33 min; Temperature: 113 °C.	61.17% of the protein extraction yield of the spent coffee powder.	[114]
Kale stalk	Frequency: 50 Hz; Power: not informed; Time: 14.93 min; Temperature: 109 °C.	96.55% of the protein extraction yield from the cabbage stalk.	[114]
Defatted watermelon seeds	Frequency: not informed; Power: 50 W; Time: 2 min; Temperature not informed.	90% extraction compared to the conventional method.	[133]
Coconut milk	Frequency: 2.5 GHz; Power: not informed; Time: 1 min. being 3 pulses of 20 s every; Temperature: 45 °C.	19.5% increase in protein extraction in coconut milk.	[64]
Soy milk	Frequency: not informed; Power: 675 W; Time: 60–120 s; Temperature: 80 °C.	24% and 44.4% increase in extraction yield and protein content, respectively, compared to the conventional method.	[134]
Jackfruit leaf	Frequency: 42 kHz; Power: 1200 W; Time: 2, 3 or 4 min; Temperature: not informed.	In the extraction of proteins from jackfruit leaves, we obtained a content of 84.1 mg/g using conventional methods, but in MW extraction we obtained a higher concentration of 95.6 mg/g.	[65]
Cottonseed meal	Frequency not informed; Power: 900 W; Time: 6 min; Temperature: not informed.	MWs can cause conformational changes in proteins, generating free radicals or large or small molecules, thereby damaging the primary, secondary, and tertiary structure of the protein and influencing solubility, emulsification, foaming, and other functional properties.	[135]
Wheat germ	Frequency not informed; Power: 186 W; Time: 3.28 min; Temperature: not informed.	The results obtained revealed that the use of MW technology as a process intensifier device for protein extraction improved the amount of protein extracted and functional properties such as water/oil retention capacity, emulsifying capacity, digestibility solubility, gelling properties and foaming capacity.	[121]
Pineapple peel waste	Frequency not informed; Power: 300 to 600 W; Time: 40 to 50 min; Temperature: not informed.	Shorter time, higher extraction rate, lower cost), it has been found to be a captivating medium for the purpose of extraction	[136]
Tremor grains	Frequency not informed; Power: 200 to 2000 W; Time: 10 min; Temperature: not informed.	Increased extraction yield in MW pretreated samples with shorter processing time (10 min) compared to conventional (1 h).	[137]
Milk caper (*L. volemus*)	Frequency: 50/60 Hz; Power: not informed; Time: 10 min; Temperature: 20 °C.	Increased extraction rate and better functionality of proteins extracted with MWs compared to conventional extractions.	[138]
Jack bean (*Canavalia ensiformis* L.)	Frequency: not informed; Power: 800 W; Time: 120 s; Temperature: 50 ± 5 °C.	MW-assisted alkaline extraction showed a higher protein content of 86.34% with interesting gelation properties.	[139]
Silkworm (*Bombyx mori*)	Frequency: not informed; Power: 730 W; Time: 1–2 min; Temperature: not informed.	Changes in the formation of protein that improved the emulsification and foaming properties.	[69]
Red algae (*Palmaria palmata*)	Frequency: not informed; Power: 50, 100, 150, 200, 250, and 500 W; Time: 5, 10, 15, 20 and 30 min; Temperature: 45 °C.	The MW treatment (30 min) provided a protein extraction yield of 47.6 ± 3.4% with purities ~90% higher compared to the conventional method.	[140]
Foxtail millet (*Setaria italica*)	Frequency: not informed; Power: 480–960 W; Time: 30–90 s; Temperature: 30 °C.	MW significantly increased protein yield compared to conventional extraction methods, improving functional properties, solubility, foaming and emulsification properties and digestibility.	[141]
Pigeon pea (*Cajanus cajan*)	Frequency: not informed; Power: 400–700 and 960 W; Time: 60–120 s; Temperature: 21–23 °C.	MW increased the protein recovery and yield within 2 min of treatment with a consequent increase in the protein solubility.	[142]

Behere et al. [133] evaluated the extraction of proteins from defatted watermelon seeds containing 50% protein. The authors used an MW-assisted extraction process with powers of 30, 50, and 70 W; a solid-to-solvent ratio of 1:10–1:40; an application time ranging from 30 s to 8 min; and a pH of the solvent in the range of 7–10, with the effects of moisture content or preleaching. The authors also compared the results with those of ultrasound-assisted and conventional extraction. A comparative study revealed 90% protein recovery by applying only 2 min of MW at a solid-to-solvent ratio of 1:30 (*w*/*v*), with a similar yield obtained with ultrasound but for 9 min with a solid-to-solvent ratio of 1:50 (*w*/*v*). Conventional extraction reached only 72% with 25 min of processing and high use of water (solid-to-solvent ratio of 1:70 (*w*/*v*)). The functional properties of the proteins obtained with the aid of MWs were superior to those of the proteins obtained via the conventional method and comparable to those obtained with the assistance of ultrasonication.

Current studies, such as those of Karki et al. [141], revealed clearer modifications in the proteins of the Foxtail millet (*Setaria italica*) by MW treatment, in addition to a significant increase in protein yield in comparison with conventional extraction methods. The authors obtained better results in terms of the techno-functional properties of protein concentrates, particularly their solubility, foaming capacity, foaming stability, and emulsifying activity index. The hydrolysates had better foaming properties and significantly greater protein digestibility (62.57%). When applying FTIR analysis, they confirmed the close relationship of the techno-functional properties and digestibility with the structural changes in the secondary protein structure induced by MWs.

In addition to the aforementioned advantages, the MW has been applied to products with value added with recent interest, such as red seaweed (*Palmaria palmata*). Compared with the conventional extraction method, the use of 500 W for 30 min was the most effective method, resulting in a final protein extraction yield of 47.6 ± 3.4% and a concentration purity of ~2-fold greater [140].

## 5. Deep Eutectic Solvents (DESs)

Deep eutectic solvents have emerged as alternatives to the commonly used volatile organic compounds. DESs are a class of green solvents formed by two or more components, mostly a mixture of organic halide salts, such as choline chloride or zinc chloride, and a hydrogen donor compound [143]. Among the hydrogen donors are amines, amides, alcohols and carboxylic acids, which remain liquids at room temperature. The mixture of components does not form ionic or covalent bonds; rather, they interact through hydrogen bonds. Its designation “deep” is due to its ability to form a eutectic mixture with a melting point much lower than that of its individual constituents [144].

Some authors describe DESs as a subclass of ionic liquids [145,146]. However, there are differences in both the starting materials and the production mechanisms. In general, these two solvents tend to present several similar characteristics, such as high thermal stability, low vapor pressure, high ionic conductivity, high biodegradability, low toxicity, and low cost.

DESs are typically easy to prepare: they use affordable equipment and are able to minimize water demand. Some DES preparation methods can be performed with additional unit operations, including vacuum evaporation, grinding, and/or freeze-drying. However, the most commonly used preparation method involves only heating and stirring the constituents together under an inert atmosphere until a homogeneous liquid is formed [147]. As it is not necessary to add any other solvent, no traditional reaction takes place; thus, no purification steps are needed. Owing to their structure and wide polarity range, the resulting DESs tend to have a good ability to dissolve micro- and macromolecules of natural products, and this property increases the potential to extract compounds such as proteins from many different sources. Therefore, it has been considered a sustainable solvent for extraction and obtaining value-added molecules [148].

There are several DESs, and depending on their composition, they can act as stabilizers, protecting the protein structure against thermal or oxidative degradation. In other cases, interactions with solvent components can lead to modifications in the protein structure, leading to possible partial unfolding and denaturation [149].

For example, Chen et al. [150] used choline chloride and glycerol-based DESs for soy protein extraction. The authors obtained an increase in the hydrophobic interaction and the hydrogen binding properties in the deep eutectic solvent. Thus, the amount of protein extracted was 0.3462 ± 0.046 g, which was close to the value obtained via extraction with conventional methods (0.3518 g).

The characterization of the extracted soy protein revealed that, although irreversible denaturation was caused by DES extraction compared with conventional methods, the proteins extracted by DES had better heat resistance and greater hydrophobicity. Notably, the technofunctional properties of proteins can be altered according to the solvent used for extraction. The unfolding of proteins and the exposure of polar groups may result in improved solubility, whereas structural modification can alter the properties of emulsification and gel formation [151].

Yue et al. [152] prepared nine mixtures of choline chloride and butanediol isomers to extract protein from oats. They reported that the proteins extracted by the binary mixture of choline chloride-1,4-butanediol/water resulted in the highest percentage of protein extraction (55.72%). The presence of moisture in the DES increased the denaturation temperature of the obtained proteins, resulting in great stability of the oat protein against thermal processing, good foaming capacity, and improved water solubility.

A study carried out by Hernández-Corroto et al. [153] demonstrated that extracting proteins from pomegranate peel via choline chloride/acetic acid resulted in an ~2-fold greater protein content (19.2 mg protein/g peel) than did the pressurized liquid extraction method (9 mg protein/g peel). In addition, the proteins obtained via DES extraction had more preserved structures with higher antihypertensive capacities than those obtained via pasteurized liquid extraction.

Lin et al. [154] extracted proteins from bamboo shoots via different molar ratios of choline chloride-levulinic acid and compared the efficiency of the DES with that of the conventional solubilization process in alkaline media. The optimal conditions for extraction performance were a hydrogen acceptor (HBA) and hydrogen donor (HBD) molar ratio of 6, with a solid–liquid ratio of 30 mg/mL. The authors reported a yield of 39.16 ± 1.22 mg protein/g dry weight, which was significantly greater than that of conventional extraction using NaOH solution (23.88 mg protein/g dry weight). The results also suggest that bamboo processing byproducts could be an interesting source of alternative proteins.

Wahlström et al. [155] mixed carboxylate salts (potassium and sodium formate and acetate) with urea to synthesize DES for the extraction of proteins from barley grains. They reported that sodium/urea acetate (molar ratio of 1:2) extracted 79% of the protein content of the grains, producing an extract with more than 50% protein. Despite the high yield and protein content of the extract, the authors recommended the use of some components, such as urea, for food applications.

A study carried out by Liu et al. [156] also combined the use of microwave irradiation to assist in the extraction of proteins from pumpkin seeds via the use of choline chloride and polyethylene glycol. They obtained an extraction yield of 93.95% and the highest pumpkin seed protein precipitation rate (97.97%) via isoelectric coprecipitation with ethanol–polyethylene glycol.

Moldes et al. [157] investigated the application of DESs in the selective extraction of proteins from the brown seaweed *Saccharina latissima* using previously freeze-dried biomass. Several experimental plans were tested, and the highest extraction yield was obtained after 1 h of processing the biomass with the eutectic mixture of 1 betaine:2 urea at 40 °C, with the addition of 47% water to the system. They reached a protein recovery of 10.6%, comparable to that of conventional methods (bead milling), but with superior selectivity. They highlighted the sustainability of the method in recovering proteins from macroalgae efficiently.

Additionally, Ecem Bayram et al. [158] explored protein extraction from bee bread, a fermented product derived from pollen that is rich in nutrients and widely used by bees as a protein source. They studied the application of 12 different DESs with different hydrogen bond acceptor (HBA)–hydrogen bond donor (HBD) combinations. In particular, choline chloride and urea not only increased the extraction efficiency but also resulted in greater valorization of the contents of free amino acids and total phenolic compounds.

These findings reinforce the versatility of DESs in the extraction of bioactive compounds from complex natural matrices. However, some challenges are encountered when using DESs on an industrial scale. Studies have reported the complexity of recovering solvents used in extraction, thus increasing operational costs [159]. Furthermore, the regulatory agencies’ definition of acceptable solvent types for food use creates greater scalability difficulties, as the solvents must be of high purity and considered Generally Recognized As Safe (GRAS) [160,161,162]. Their application to other products is demonstrated in Table 4. In view of the previous studies presented in Table 4, the use of DES as a nonconventional technology becomes a viable solution to reduce the impact of conventional protein extraction from alternative sources. Potential intensification of the process, lower environmental impact, ease-to-prepare aspects and the ability to preserve or even improve certain techno-functional properties of the extracted proteins, such as solubility and thermal resistance, could be observed.

However, compared with ultrasonic, microwave, and pulsed-electric methods, the use of DESs presents greater challenges and limitations, such as the potential for denaturation of the extracted proteins depending on the composition of the DES and the extraction conditions [163]. In addition, the solvent components need to be carefully selected to avoid undesirable residues, especially in food applications. There is a lack of standardized methods for different matrices, which requires additional and specific studies to ensure the replicability of the results and evidence the improvement over other alternative technologies. Under suitable conditions, DESs represent a potential technology for scaling up protein extraction in industrial applications [164,165].

**Table 4:** Recent studies on the improvement of deep eutectic solvents in the extraction of alternative proteins.

Product	Extraction Conditions			Observed Effects	References
	Solvent	Temperature (°C)	Time (min)		
Oats	Choline chloride-butanediol	80	90	The presence of water from DES increased the denaturation temperature, denoting great stability of the protein against thermal processing and foaming capacity. The presence of water improved the solubility of the protein.	[152]
Soy	Choline-glycerol chloride	60	234	Positive hydrophobic and hydrogen bonding interaction in DES. Irreversible denaturation was caused by DES extraction during heating. Extracted protein showed better heat resistance and stronger hydrophobicity.	[150]
Bamboo shoots	Choline chloride-levulinic acid	80	50	Protein extraction yield of 39.16 mg/g significantly higher than the conventional sodium hydroxide extraction method (23.88 mg).	[154]
Brewer’s Spent Grain	Carboxylate-urea	80	240	79% extraction yield was obtained with >50% protein.	[155]
Pomegranate peel	Choline chloride-acetic acid	120	15	19.2 mg/g of protein were obtained. The hydrolysis obtained from proteins extracted by DES showed high antihypertensive capacity.	[153]
Pumpkin seed	Choline chloride-polyethylene glycol (PEG)	43	4	The extraction yield was 93.95% and the protein precipitation rate was 97.97, with a precipitation time of only 4 min using these conditions.	[156]
Cold-pressed cakes produced from linen (*Linum usitatissimum*)	Choline chloride and glycerol	80	60	In general, the proteins were extracted with DES and precipitated with water, obtaining higher yield. An improvement in extraction was achieved by increasing the temperature of the treatment. Different acylglyceride profiles were identified in the residual oils obtained by solvent extraction of the cakes.	[159]
Cold-pressed cakes produced from camelina (*Camelina sativa*)	Choline chloride and glycerol	80	60	An improvement in extraction was achieved by increasing the temperature of the treatment. Different acylglyceride profiles were identified in the residual oils obtained by solvent extraction of the cakes.	[159]
Cold-pressed cakes produced from sunflower (*Helianthus annuus*).	Choline chloride and glycerol	80	60	An improvement in extraction was achieved by increasing the temperature of the treatment. Different acylglyceride profiles were identified in the residual oils obtained by solvent extraction of the cakes.	[159]
Sesame meal flour	Choline chloride: 1,3-Propanediol 1:2	60	60	The results showed that the purity of the protein extracted with DES was significantly higher (up to 93%) than that with the precipitation extraction method (77.1%).	[149]
Oats	Choline chloride: 1,3-Propanediol 1:2	80	90	Higher protein content—62.50%; with extraction yield of 8.18% and protein recovery—35.76%	[94]
Oats	Choline chloride: ethylene glycol 1:2	55	45	Overall, protein extraction and recovery yields were higher for oat proteins extracted by hydrated DESs. DESs demonstrated their great potential for biorefining oat proteins obtained from biscuit flour.	[152]
Rapeseed Cake	Choline chloride: glycerin 1:2	100	120	The precipitate yield improved with the increase in the treatment temperature, reaching a maximum of 20% and 35% at 140 °C. In general, the protein content of the extracts was 40–50%, which is up to 20% more than the starting materials. Extraction yield—9%.	[162]
Soy	Choline chloride: glycerol	80	234	Extraction yield—0.3462 and precipitation yield—0.3192.	[162]
*Punica granatum*	Choline chloride: glucose 1:1	80	234	Single extraction of pomegranate seed proteins using DES under alkaline conditions allowed for to extraction of up to 15.3 g protein/100 g pomegranate seeds (61% protein in pomegranate seeds). Extraction yield—4.2 g/100 g.	[153]
*Punica granatum*	Choline chloride: acetic acid 1:2	60	15	Single extraction of pomegranate seed proteins using DESs under alkaline conditions allowed to extract up to 15.3 g protein/100 g pomegranate seeds (61% protein in pomegranate seeds). Extraction yield—4.2 g/100 g.	[64]
Fava bean	Choline chloride: glycerol.	50	60	DES resulted in higher rate and yield of protein extraction. The secondary structures of the obtained concentrates revealed an increase in the α-helix content (21.37%) in the proteins extracted with DES compared to proteins extracted using the conventional method (10.68%).	[166]
Seaweed (*Saccharina latissima*)	Betaine:2Urea:Water	30	60	Good protein recovery yield (11%), higher selectivity, and the suitable particle size of extracted proteins (5–10 kDa).	[157]
Mushroom stem (MS)	1choline chloride:2 glycerol and choline chloride: lactic acid	50	60	DES efficiently extracted proteins from lignocellulosic biomass with higher rate of protein recovery compared to alkaline solubilization. DES also showed better preservation of the emulsion capacities and balanced foaming properties, with enhanced foaming properties.	[167]
Cyanobacterial biomass (*Spirulina*)	Choline chloride and urea	25–30	15–45	Together with US, DES solubilization achieved a high protein yield (80.62%). The techno-functional properties of the extracted proteins, including high foaming capacity, emulsifying capacity, digestibility, and antioxidant properties, were highlighted.	[168]
Bee bread (Perga)	Choline chloride and urea.	35	25	Extracts obtained with DES showed higher levels of total protein, total individual phenolics, and total individual amino acids in comparison to ethanol solubilization.	[158]
Canola/rapeseed (*Brassica napus*)	ratio of choline chloride/d-sorbitol, glycerol, d-glucose, or urea/water.	35	25	All studied DES extracted higher percentages of napins (small and water-soluble proteins), resulting in more preserved protein structures compared to alkaline treatments.	[169]

Although ultrasound, pulsed electric fields, microwaves, and deep eutectic solvents represent the most studied nonconventional technologies for protein extraction, other methods have also gained attention due to their complementary roles in the production process. (Re)emerging techniques, such as reverse micelles and aqueous two-phase systems, are not primarily aimed at maximizing extraction yield but rather at improving the partitioning, purification, or selective recovery of protein fractions. Therefore, rather than competing directly with the aforementioned emerging technologies, these approaches can act as follow-up or parallel strategies, enabling greater purity, functionality, or efficiency in protein extraction processing. Therefore, it is relevant to address these alternative methods and their potential in the context of protein extraction.

## 6. Other Emerging Technologies

In addition to nonconventional technologies, other emerging (or reemerging) practices have shown the potential to be employed solely or in combination with nonconventional technologies to increase the viability of protein extraction from sustainable sources. Among these methods, extraction via reverse micelles has become an interesting technique for the efficient recovery of proteins from alternative sources.

The technique of reversing micelles consists of a micelle-type system but contains an aqueous inner core stabilized by nanosized surfactants, making the solubilization of hydrophilic compounds possible in nonpolar media [170]. In the case of protein extraction, the soluble proteins are initially solubilized into the aqueous inner core of the reverse micelles (forward extraction) for further recovery by breaking down the micelle structure in a second extraction (backward extraction). The most common surfactant used for reverse micelle preparation is anionic AOT (sodium bis (2-ethylhexyl) sulfosuccinate), which is generally recognized as safe (GRAS). Conventional backward extraction is performed by adding an equivalent volume of aqueous solution to the forward extraction solutions [24].

The advantages of this method over conventional extraction are the possibility of recovering both the surfactant and the solvent; it has a low associated cost; it is scalable; and the extracted protein is preserved in the micelles, and, thus, denaturation is minimized [24]. When dealing with oily but protein-rich products such as seeds, reverse micelles have advantages over conventional extraction methods for simultaneously fractioning oil and proteins, as the presence of lipids increases the difficulty of protein recovery.

Therefore, studies have aimed to use reverse micelles for proteins (either with lipids or without lipids) from sustainable sources, such as hemp seeds [170], cruciferous oilseeds [171], walnuts [172], tea byproducts [173], and plant sources in general [174]. In general, proteins extracted by reverse micelles tend to present more preserved techno-functionality, a more compact structure, and greater solubility than those extracted by conventional methods. An analysis of the previously cited studies revealed that the yield of protein extraction in the forward step was greater than 50%, with more than 80–90% recuperation from the backward solution. Moreover, the method used for backward extraction (the recovery of proteins from the inner core) plays an important role in determining their final properties. For this purpose, nonconventional technologies such as microwaves [172] and ultrasound [173] can be incorporated into the process.

Another re-emerged technique is the use of an aqueous two-phase system (ATPS), which is currently used for recovering proteins from sustainable sources and even more for purifying them. In this type of liquid–liquid extraction, two immiscible compounds are put in contact. Generally, a hydrophilic polymer (e.g., polyethylene glycol, dextran, or polypropylene glycol) and a salt (e.g., phosphate, sulfate, or citrate), or even a mixture of two polymers, are used. Owing to the low interfacial tension, the diffusion of solvents and compounds of interest is promoted between the biphasic system [175]. The factors affecting the partitioning of proteins are related to the molecular weight and concentration of the polymers, ionic strength of the salt, pH of the solution, and additives used to alter hydrophobicity [176]. It is worth noting that some limitations prevent its use on an industrial scale, mainly concerning cost, polymer/salt recovery, and purification aspects [177,178]. Like reverse micelles, ATPSs have been continuously developed in combination with nonconventional techniques, such as DESs [179] and ultrasound [180], to improve fractionation.

The main advantage of using ATPS for protein extraction is related to the fact that denaturation or loss of biological activity cannot easily occur, thus tending to preserve the functionality and structure of ATPS [179]. In addition, varying the molecular weight of polyethylene glycol and the type of saline solution can cover a wide range of proteins to be selectively concentrated and separated from other compounds [181].

Studies on the use of ATPSs for protein extraction from microalgae have reported their suitability because of their ability to separate biomolecules (sugars, pigments, and proteins) [182]. The extraction of polysaccharides and proteins from *Pleurotus ostreatus* was studied by Yang et al. [183], who also used ethanol/K_2_HPO_4_ as ATPS. The authors obtained more than 75% protein recovery from the fungus. Compared with alkaline solubilization, for example, ATPS resulted in greater efficiency and purity and better preservation of the physical structure.

When alternative sources with potential for large-scale production are evaluated, Gu et al. [184] reported that ATPS is suitable for protein extraction and purification from corn extracts. These authors emphasized that the concentrations of polyethylene glycol and Na_2_SO_4_, together with the ionic force (by NaCl addition), can be used to recover either hydrophobic or hydrophilic proteins. An ATPS consisting of betaine-urea as a DES together with K_2_HPO_4_ was used for extracting proteins from walnut and its corresponding cold-pressed meal. Compared with alkaline solubilization and isoelectric precipitation, the obtained proteins presented better color and lower surface hydrophobicity, with distinct characteristics of superior solubility at low pH [185].

In addition to the possibility of incorporating nonconventional technologies into these techniques, the addition of pretreatments or further steps may be a potential alternative. Saw et al. [186] used a liquid biphasic flotation (LBF) system when extracting proteins from *Persicaria tenulla* leaves. An antisolvent step, in which surface-active or hydrophobic compounds are adsorbed on bubble surfaces and then recovered by flotation in a superior organic solution, is added.

## 7. Comparisons and Current Industrial Challenges

After detailing each unconventional technology, it is crucial to make a critical comparison among them based on the presented studies. Ultrasound (US), for example, has been shown to be highly effective for dense plant matrices; however, its processing on an industrial scale is limited by high energy consumption and additional cooling costs. [187,188,189]. US also requires high energy and is not always industrially scalable [190]—demanding physical assessment regarding wave resonance in the reactor and probe disposal in the batch chamber. Continuous processes are also challenging in order to match the efficient exposure time with the processing needs. In addition to its costs, if not carefully controlled, it can lead to thermal degradation of sensitive compounds present in the samples [191].

On the other hand, pulsed electric fields (PEFs) operate with lower energy consumption compared to US, offering potential for microalgae and biomass, considered more fragile materials [192,193]. However, electrode durability poses a challenge for scalability, as it generates high equipment costs Differently to US, the scalability of [187,188,189] continuous PEF treatment is easier to design due to the short treatment time and lower residence time in the treatment chamber. Microwave-assisted extraction (MW) was reported to provide rapid and efficient protein recovery, but increasing the frequency leads to challenges such as heating uniformity [194,195]. The review published by [196] reinforces that studies are still incipient when evaluating high amounts of raw material (~0.5 kg/batch). They also highlight that, in contrast, the use of deep eutectic solvents (DESs) does not entail additional challenges other than the recovery and reuse of the solvent.

Deep eutectic solvents (DESs) have proven to be a sustainable alternative to conventional solvents, enabling protein recovery while preserving their functionality. However, the cost of solvents and recovery procedures limits their industrial viability. Furthermore, there are no methodologies to verify the toxicity of DES [191,196]. Overall, US and MW are closer to industrial use and are reported to be of low environmental impact [197]. However, PEF and DES remain in the early stages, requiring further study. Other aspects than the technology chosen itself are the management and application of the raw material for extraction. The use of wet sources may avoid the drying process, thus demanding low energy and water consumption. On the other hand, the extraction efficiency tends to be damaged in these applications. Therefore, the selection of technology must be specific to each case, considering the type of substrate, the desired functionality, and energy efficiency.

## 8. Conclusions

Nonconventional extraction methods have varying impacts on the characteristics and quality of extracted proteins, mainly when dealing with different alternative sources. These methods represent alternatives to intensify and implement extraction from alternative sources, especially for obtaining plant-based proteins and promising blue proteins (from aquatic sources) and concentrates/isolates from insects and agroindustrial byproducts.

Certainly, nonconventional technologies offer benefits and challenges that should be considered when choosing the technology and the protein source. Ultrasound-assisted extraction, for example, stands out for its efficiency in breaking up dense cell structures, reducing the average size of proteins, and increasing surface area and roughness, which improves properties such as solubility, emulsification, and the ability to favor the Maillard reaction.

On the other hand, the pulsed electric field (PEF) tends to be highly applicable in plant sources and microalgae, providing high yields but also preserving sensory and techno-functional properties according to the processing parameters. The microwave application, in turn, combines efficiency, high mass transfer rates, and lower environmental impact and is able to preserve the functionalities of proteins when used under controlled and fast conditions, even though there is still a risk of denaturation under inappropriate parameters. Deep eutectic solvents (DESs) have greater potential for innovation but also present significant challenges, such as the choice of suitable solvent composition and combination, which makes standardization for different matrices difficult. However, once the method is well established, the technology can be easily scaled up. Techniques such as reverse micelles and aqueous two-phase extraction have also been observed as complementary approaches not only for extracting proteins but also for purifying them for further use.

In conclusion, compared with conventional alkaline solubilization, all of these covered technologies have the potential to improve protein extraction and reduce the environmental impact (possible reductions in the use of chemicals, water consumption, temperature, etc.). Owing to the focused source of protein, each one may present some specificities. When the process is properly designed, nonconventional technology offers sustainable and improved methods for recovering proteins from nonanimal sources, highlighting new possibilities for future industrial applications.

## Data Availability

No new data were created or analyzed in this study. Data sharing is not applicable to this article.
